# Effect of Cu Doping on Synthesis, Composition and Sensor Properties of In_2_O_3_ Nanostructures

**DOI:** 10.3390/nano15120925

**Published:** 2025-06-14

**Authors:** Mariya I. Ikim, Elena Yu. Spiridonova, Olusegun Johnson Ilegbusi, Leonid I. Trakhtenberg

**Affiliations:** 1 N.N. Semenov Federal Research Center for Chemical Physics RAS, 4 Kosygin Street, Moscow 119991, Russia; ikimmary1104@gmail.com (M.I.I.); litrakh@gmail.com (L.I.T.); 2 Department of Mechanical and Aerospace Engineering, University of Central Florida, Orlando, FL 32816, USA; 3 Chemical Faculty, Lomonosov Moscow State University, Moscow 119991, Russia

**Keywords:** indium oxide, nanostructured composite, hydrothermal synthesis method, cubic and rhombohedral structures, particle size, conductivity, sensor response, hydrogen, carbon monoxide

## Abstract

Cu-doped In_2_O_3_ nanocomposites with copper compositions of 1–3 wt.% are synthesized by a hydrothermal method using water or alcohol as a solvent. Cubic In_2_O_3_ is formed when water is used for synthesis, while composites synthesized in alcohol contain rhombohedral In_2_O_3_. This trend is independent of the amount of copper introduced. The Cu ions are shown to be uniformly distributed in the In_2_O_3_ nanoparticles without significant destruction of the indium oxide structure. All the composites exhibit a porous structure that depends on the solvent used for the synthesis. The addition of copper to both crystalline forms of indium oxide increases the resistance of the films and reduces the operating temperature. The phase state of indium oxide also affects the conductivity of the composites. There is an increase in sensory response to H_2_ and CO with the introduction of Cu into samples with cubic structure, but a reduction in response in samples with the rhombohedral phase of indium oxide.

## 1. Introduction

Metal oxides, such as ZnO, SnO_2_, WO_3_ or In_2_O_3_, are common materials used for the sensitive layer of modern sensors capable of effectively detecting various gases [1,2,3,4,5]. Indium oxide is a semiconductor with a relatively high concentration of conduction electrons and unique electrical and optical properties. It is a wide-bandgap *n*-type oxide with high conductivity and a large number of surface defects [6]. It has two typical crystal structures: the stable body-centered cubic structure of bixbyite and the metastable rhombohedral structure of corundum [7,8,9].

Single-component In_2_O_3_ sensors have certain inadequacies, such as high operating temperature, insufficient sensitivity, and low selectivity, which limit their practical application. A variety of strategies are therefore often used to improve the sensory characteristics of indium oxide, including the creation of heterostructures, the addition of noble metal nanoparticles and doping with metal ions [10,11,12,13,14,15]. The introduction of ions of different valences and sizes into the structure of indium oxide is a promising method, which results in a decrease in the size of nanoparticles and an increase in the number of defects in the crystallites. In addition, foreign ions can act as donors or acceptors of free electrons in the sensitive layer, influencing the characteristics of charge transfer.

When metal oxides are isovalently doped with metal ions of a larger size than the main ions of the lattice, the concentration of oxygen vacancies increases. This leads to an increase in the sensory effect, which is clearly seen in the example of composites where In^3+^ ions (0.80 Å) are replaced by La^3+^ ions (1.03 Å) [16,17].

During heterovalent doping, not only do deformations occur but also new charges arise in the structure due to the difference in valences of metal ions. Thus, the addition of Co ions increases the number of oxygen vacancies, which changes the electron concentration in the conduction band and ultimately improves the sensor performance [18,19,20]. The redox reaction between Co^2+^ and Co^3+^ results in the formation of such vacancies, which affects the diffusion of oxygen ions and enhances the adsorption of the target gas, thereby allowing more gas molecules to react [18]. In addition, the introduction of cobalt has a beneficial effect on several sensor characteristics, including a decrease in the crystallite size, a narrowing of the band gap and a shift in the Fermi level [19].

The response of In_2_O_3_ sensors to formaldehyde decreases with increased valence of the additives Zn^2+^, Sb^3+^, Zr^4+^ and Nb^5+^. The largest response is observed for acceptor doping with zinc ions [21]. For example, the introduction of 3 mol.% Zn enhances sensitivity to triethylamine at a fairly low temperature of 140 °C, which is attributed to the starfish-like morphology with a higher specific surface area caused by doping [15]. Defects created by introducing 1% Zn into the indium oxide structure increase the surface adsorption capacity, resulting in a two-fold increase in the response to NO_2_ under UV irradiation [22]. Zn-In_2_O_3_ films exhibit higher resistance due to an increase in the barrier height in the intergranular regions [23]. Copper doping also increases the resistivity of indium oxide films because Cu is an electron acceptor [24]. Doping of In_2_O_3_ with divalent copper at a concentration of 1 mol.% increases the sensor response to NO_2_ by approximately 14.5 times compared to undoped In_2_O_3_ due to the possible catalytic effect of Cu ions [25]. Note that the introduction of different concentrations of Cu into In_2_O_3_ can significantly affect the sensitivity to H_2_S [26]. In addition, the dopant, regardless of its valence, can cause phase transformations in the In_2_O_3_ structure, which in turn affects the electrophysical properties [27,28,29].

The above studies focused on effects of the valence of doping additives on the sensory characteristics of indium oxide, as the role of metal ion additives in different crystalline modifications of In_2_O_3_ was rarely considered [30]. In this study, nanocomposites based on cubic or rhombohedral indium oxide with different concentrations of copper ion dopants are synthesized using the hydrothermal method. The effects of both the In_2_O_3_ phase and the Cu content on the structure, conductivity and sensor properties in the detection of H_2_ and CO are investigated for the first time.

## 2. Experiments Performed

Indium oxide and composites containing 0.5 to 3 wt.% copper were synthesized by the hydrothermal method [30,31,32]. First, 2 mmol of In(NO_3_)_3_·4H_2_O (>99.5% purity) and 18 mmol of urea were added to 80 mL of distilled water or ethyl alcohol and stirred vigorously. The resulting solution was then kept in an ultrasonic bath at a temperature of 30 °C for 1 h. Next, the mixture was placed in a 100 mL Teflon autoclave for hydrothermal treatment and heated for 3 h at 160 °C. After natural cooling to room temperature, the resulting precipitate was separated in a centrifuge, washed several times with water and dried at 90 °C. The product was heated at a rate of 15 °C/min to 500 °C and maintained at this temperature for 2 h. Copper ion-doped In_2_O_3_ nanomaterials were prepared by adding different amounts of Cu(NO_3_)_2_·6H_2_O to the above aqueous or alcoholic solutions.

In order to form sensor films, the synthesized composites in the form of homogeneous aqueous suspensions were applied to polycor plates equipped with a platinum heater and contacts. The detailed procedure for the formation of films and the study of their electrophysical and sensory characteristics are described in a previous study [33].

X-ray phase analysis of the composite systems obtained was performed using the Rigaku Smartlab GP X-ray diffractometer(Japan) (CuKa radiation with a wavelength of 1.5406 Å). The specific surface area and pores were determined using the low-temperature nitrogen adsorption method on a NOVA Series 1200e Quantachrome device (USA). The structure of the nanoparticles and the distribution of metal ions in the composites were determined by TEM and EDX methods using a Tecnai Osiris FEI instrument(USA) equipped with an energy-dispersive analysis system. The electronic structure of metal ions on the surface of the nanoparticles in the composite was recorded on a Prevac EA15 System spectrometer(EU) using Al(Kα) radiation (1486.6 eV) as an excitation source. All binding energies were referenced to the C 1s peak at 284.8 eV.

The sensory response of the composites was measured in the temperature range of 300–520 °C at an air humidity (RH) of 30%. Commercially certified gas mixtures containing 0.9% H_2_ or CO were used for the measurements. The sensor response S is defined as S = R_0_/R_g_, where R_0_ and R_g_ are the resistances of the composites in clean air and in air containing the analyzed gas, respectively, and was recorded using a Keysight digital multimeter.

## 3. Results and Discussion

### 3.1. Structural Characteristics of Cu-Doped In_2_O_3_ Nanocomposites

**Figure 1:**
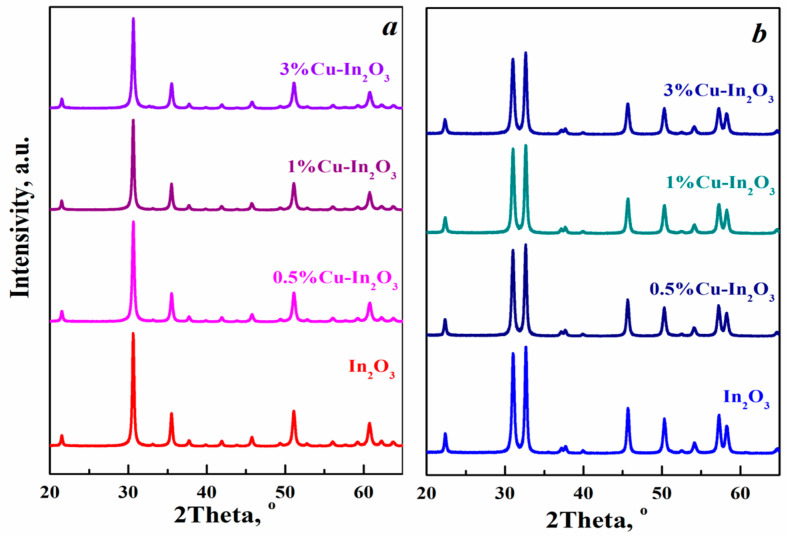
XRD spectra of Cu-In_2_O_3_ composites synthesized by the hydrothermal method using aqueous (**a**) and alcohol (**b**) solutions of indium and copper nitrates.

The phase composition of the composite materials obtained was determined by the XRD method. The only peaks recorded in the XRD spectra of composites synthesized from aqueous solutions of indium and copper nitrates are those with a cubic structure of bixbyite In_2_O_3_ with preferred orientations (222) and (400) (Figure 1a). All the diffraction peaks in the spectra of composites synthesized from alcohol solution of the corresponding salts belong to the rhombohedral phase of indium oxide of the corundum type with preferred orientations (104) and (110) (Figure 1b). The phase composition of indium oxide depends on the solvent used in the synthesis. During a hydrothermal process, the choice of solvent influences the formation of one or another intermediate product. Both In(OH)_3_ and InOOH can be formed probably due to the difference in pH of the solutions used [34]. Subsequent annealing of the cubic modification In(OH)_3_ leads to the formation of a body-centered cubic phase of indium oxide, and the orthorhombic form of InOOH produces rhombohedral indium oxide [7,8,9].

Diffraction peaks from other impurities are not observed, indicating the purity of the synthesized samples. The large intensity of diffraction peaks in the XRD spectra indicates that the systems investigated have high crystallinity.

No other crystalline phase or obvious impurity peaks are detected in the spectra of samples containing different amounts of copper, indicating that the crystalline phase of copper oxides is not formed during the synthesis. This is observed with the introduction of up to 3% Cu into the color-like In_2_O_3_ microspheres synthesized by the hydrothermal method [25]. It was shown that in *x*% Cu-In_2_O_3_ (*x* = 2, 4, 6, 8) nanofibers obtained using a combination of electrospinning and calcination processes, the crystalline phase of CuO is formed upon the introduction of more than 6% Cu ions [26]. This result implies that CuO is incorporated into the In_2_O_3_ lattice without changing the structure of indium oxide (Figure 1). It is also worth noting that the introduction of La, Fe or Ce ions, for example, into the initial mixture of polymorphic modifications of indium oxide leads to an increase in the composition of the rhombohedral phase [35].

The diffraction peaks (222) for cubic and (104) for rhombohedral indium oxide in Cu-doped composites are shifted towards higher 2θ values compared to pure In_2_O_3_ phases (Table 1). The shift of the peaks occurs due to the difference in the radii of the Cu^2+^ (0.73 Å) and In^3+^ (0.81 Å) ions when copper ions are introduced into different indium oxide lattices. In addition, the smaller ionic radius of copper results in lattice compression of both cubic and rhombohedral indium oxide (Table 1). A similar phenomenon was previously observed in samples based on rhombohedral indium oxide doped with up to 7.5% Zn [36].

The size of nanoparticles in composites was estimated using the Debye–Scherrer equation. A broadening of the peaks is observed upon the addition of copper, regardless of the phase state of In_2_O_3_. The average nanoparticle size decreases with enhanced copper concentration in the composites (Table 1). The decrease in crystallite size is associated with the deformation of the indium oxide lattice because of the introduction of copper.

**Figure 2:**
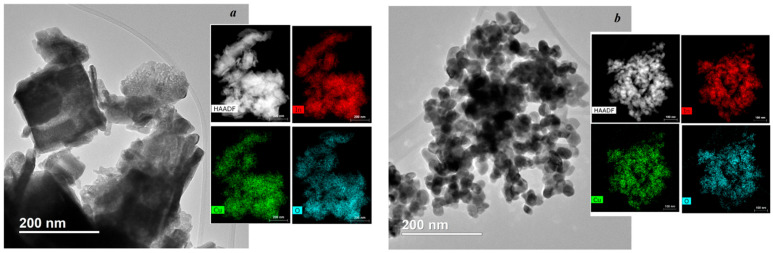
TEM, HAADF and energy dispersive analysis of 3% Cu-In_2_O_3_ composites synthesized by the hydrothermal method using aqueous (**a**) and alcohol (**b**) solutions of indium and copper nitrates.

According to TEM data, hydrothermal samples synthesized from aqueous solution are agglomerates of rectangular-shaped nanoparticles of non-uniform size (see Figure 2a). Nanoparticles in composites with a rhombohedral structure have a nearly spherical shape and are uniform in size (see Figure 2b). The nature of the solvent used during the hydrothermal reaction affects not only the crystal structure of indium oxide but also its morphology [37]. For example, ether has been shown to promote the formation of nanofibers, while other solvents used in the hydrothermal process, such as acetone and ethylenediamine, result in spherical nanoparticles [38]. Thus, the generation of In(OH)_3_ can lead to the formation of particles with a rectangular shape, and in the case of InOOH—spherical particles. The amount of water during the synthesis of In_2_O_3_ nanostructures with the addition of Zn influenced their crystalline structure, which, in turn, determined the shape of the particles [39]. The spatial distribution of In, O and Cu was determined using energy-dispersive mapping of the corresponding elements in the composites (see Figure 2). In addition to the elements In and O, the element Cu is observed with uniform dispersion, which indicates a uniform distribution of copper in In_2_O_3_ nanoparticles regardless of its phase. The TEM, energy dispersive and X-ray diffraction data are all in good agreement.

**Figure 3:**
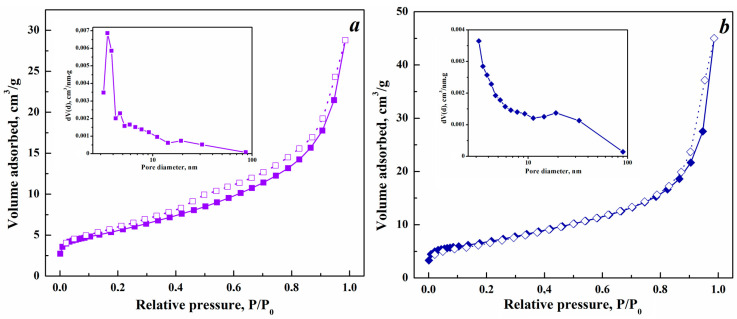
Isotherms of adsorption (filled symbols) and desorption (empty symbols) of nitrogen at 77 K of 3% Cu-In_2_O_3_ composites synthesized by the hydrothermal method using aqueous (**a**) and alcohol (**b**) solutions of indium and copper nitrates. (insets are the corresponding pore size distribution plots).

The adsorption–desorption isotherms of the composites obtained, regardless of the phase state of indium oxide and the concentration of copper introduced, have a type IV shape with a hysteresis loop H3 according to the IUPAC classification, which indicates the mesoporous nature of the synthesized systems (Figure 3). However, the shape of the hysteresis loop is influenced by the crystalline phase of In_2_O_3_, implying a difference in the mesoporous structure of the composites. For samples with cubic structure, the hysteresis closes in the region of relative pressures P/P_0_ of about 0.4 (see Figure 3a), in contrast to systems with rhombohedral lattice for which the hysteresis closes at P/P_0_ of approximately 0.8 (see Figure 3a).

The specific surface area of the samples was determined using the BET method in the range of relative pressures P/P_0_ = 0.05–0.3 (Table 2). The specific surface area of copper-containing composites is greater than that of pure In_2_O_3_ and does not depend on its structure (Table 2). The mesoporous structure of the samples was studied using the BJH method for the desorption branch of the isotherm for relative pressures P/P_0_ in the range of 0.35 to 0.99, i.e., for pores with a diameter of more than 3 nm. The pore size of samples synthesized from an aqueous solution of the corresponding salts is in the range of 3.5–3.9 nm and is essentially independent of the copper concentration (see Table 2 and inset in Figure 3a). Samples synthesized from alcohol solution are characterized by pores with a diameter of 3.2 nm, and 19–20 nm (see inset in Figure 3b). The pore size also decreases with an increase in copper concentration (Table 2). Note a slight increase in the mesopore area and volume, as well as the total pore volume, with an increase in the Cu concentration, and these values are independent of the indium oxide phase. The net result is an increase in the specific surface area of SBET (Table 2). In general, a higher specific surface area provides more active sites for adsorption, diffusion and reactions with gases [40].

**Figure 4:**
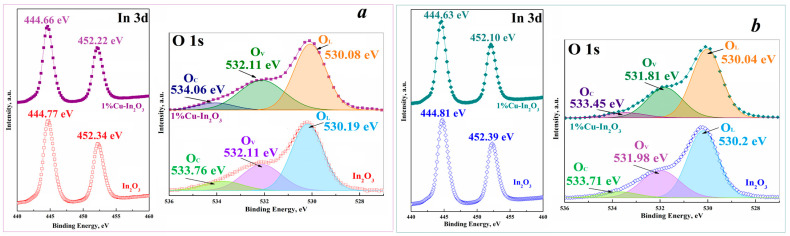
High-resolution XPS spectra of In 3d and O 1s: (**a**) In_2_O_3_ and 1% Cu-In_2_O_3_ synthesized from aqueous solutions of indium and copper nitrates, (**b**) In_2_O_3_ and 1% Cu-In_2_O_3_ synthesized from alcohol solutions.

The elemental composition and valence states of the ions in the synthesized materials were analyzed using XPS. Peaks characteristic of the elements In, O, Cu and C were detected in the XPS spectra. The peak from C 1s refers to the internal standard. The observed weak peak of copper is due to its lower concentration compared to other elements. The concentrations of Cu 2p ions relative to O 1s, In 3d and C 1s in the composites are 0.46, 0.93 and 2.86 at.%, respectively, which are quite close to the ratio during synthesis. The valence states of the elements were further analyzed using high-resolution spectra (see Figure 4). Two peaks are observed at 452 eV and 444.6 eV in the high-resolution spectra of the In element, corresponding to the spin-orbit energy states of In 3d3/2 and In 3d5/2, respectively (Figure 4a,b). This result indicates that the valence state of indium in the samples corresponds to 3^+^. In addition, the location of these peaks is shifted towards lower binding energy compared to undoped samples. This is evidence of the interaction between Cu and In as a consequence of the incorporation of Cu atoms into the In_2_O_3_ lattice. The high-resolution spectrum of O1s can be resolved into peaks at 530 eV, 532 eV and 534 eV, which are attributed to lattice oxygen (O_L_), oxygen vacancies (O_V_) and chemisorbed oxygen (O_C_), respectively (see Figure 4a,b). The relationship between the O_V_, O_C_ and O_L_ peaks can be further used to evaluate the sensory response in gas detection (Table 3).

### 3.2. Conductivity and Sensory Properties of CuO-In_2_O_3_ Composites in Detection of H_2_ and CO in Air

**Figure 5:**
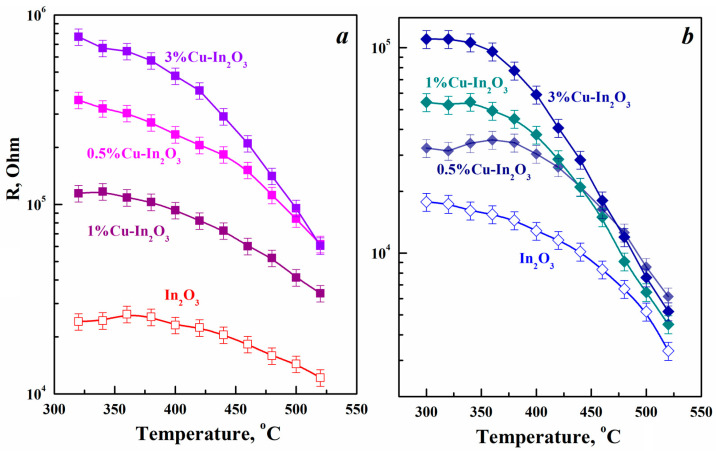
Temperature dependences of the resistance of Cu-In_2_O_3_ composites synthesized by the hydrothermal method using aqueous (**a**) and alcohol (**b**) solutions of indium and copper nitrates.

The characteristics of the change in temperature dependence of the conductivity of sensor films based on In_2_O_3_ and Cu-In_2_O_3_, which have different crystalline phases of indium oxide, are largely the same. In the range from 300 to 550 °C, the resistance of all synthesized films gradually decreases with increasing temperature, which is typical for *n*-type semiconductors (Figure 5). The activation energy of conductivity (*E*) was determined both in air and in the presence of the target gas from the data obtained. The formula *G* = 1/*R* = *G*_0_exp[−*E*/*k_B_T*] was used in this case, where *G* and *R* are the conductivity and resistance of the samples, respectively, *G*_0_ is the pre-exponential factor, *T* is absolute temperature and *k_B_* is Boltzmann constant. The *E* values for each sample were obtained by linearly fitting the slope of the ln*R* line versus 1/*T* using the data presented in Figure 5. The introduction of copper into different polymorphic modifications leads to an increase in the activation energy for rhombohedral indium oxide from 0.48 to 1.05 eV in air and from 0.38 to 0.56 eV in hydrogen. The corresponding values for the cubic phase are 0.32 to 0.6 eV in air and 0.53 to 0.65 eV in hydrogen. Therefore, the introduction of copper, regardless of the In_2_O_3_ phase, causes an increase in the potential barrier that prevents the movement of charge carriers, which leads to increases in the film resistance. In addition, the incorporation of copper into the In_2_O_3_ structure was established using XRD, TEM and EDX methods. Cu^2+^ ions either replace In^3+^ ions or are introduced into the lattice interstices. In the case of substitution, the resulting holes will recombine with electrons, which are the main charge carriers in In_2_O_3_.

Structural parameters also influence the conductivity of the composites, which may be due to the decrease in the size of In_2_O_3_ nanoparticles with increasing copper concentration in such systems (see Table 2). It has been reported that the resistance of oxide semiconductors increases with decreasing grain size [41]. The above indicates that the increase in resistance is due to volume effects, such as temperature, defects formed due to alloying, and particle size.

**Figure 6:**
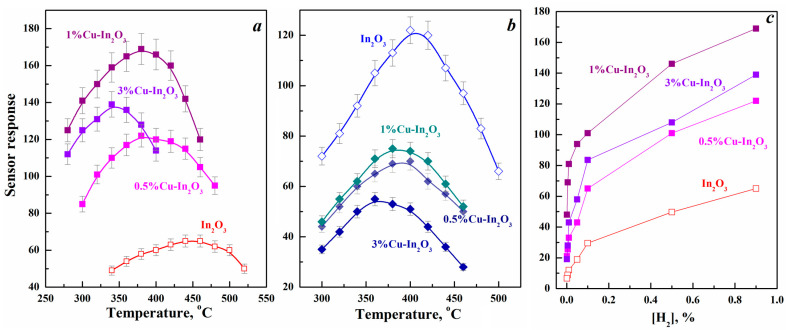
Temperature dependences of the sensory response to 0.9% H_2_ of Cu-In_2_O_3_ composites synthesized by the hydrothermal method using aqueous (**a**) and alcohol (**b**) solutions of indium and copper nitrates. Dependence of sensory response on H_2_ concentration of Cu-In_2_O_3_ composites synthesized by the hydrothermal method using aqueous solution (**c**).

The temperature dependence of the sensor response to 0.9% H_2_ is characterized by the presence of a maximum that shifts to a lower temperature with increasing copper concentration (Figure 6).

As the temperature increases, the rate of chemical reaction of the analyzed gas on the sensor surface increases, but the amount of adsorbed gas decreases due to its increased rate of desorption. The competition of these two processes explains the presence of a maximum in the temperature dependence of the sensory response [10]. The introduction of copper into indium oxide leads to a decrease in the operating temperature regardless of its phase state, which is associated with a decrease in the activation energy of the reaction between the molecules of the detected gas and negative oxygen ions. Note that an increase in the concentration of copper ions in cubic indium oxide results in a decrease in temperature by 100 °C, while the corresponding decrease in rhombohedral indium oxide is only 40 °C.

The sensory response to hydrogen increases regardless of the detection temperature in composites synthesized from aqueous solutions of indium and copper nitrates for all the copper concentrations considered. At the same time, the composites synthesized from alcohol solutions of the corresponding salts exhibit the opposite result. This trend is probably due to the difference in changes in morphological and structural parameters with the introduction of copper into the cubic and rhombohedral phases of In_2_O_3_. In addition, the dependence of sensitivity on hydrogen concentration was obtained for composites with cubic In_2_O_3_ structure (see Figure 6c). With a gradual increase in concentration from 10 ppm to 0.9% H_2_, the composite containing 1% Cu produces a much faster response in the range of 10–100 ppm hydrogen. It can be concluded that doping of cubic phase indium oxide by 1% copper not only results in an increase in response to hydrogen at a lower temperature but also provides a lower detection limit.

**Figure 7:**
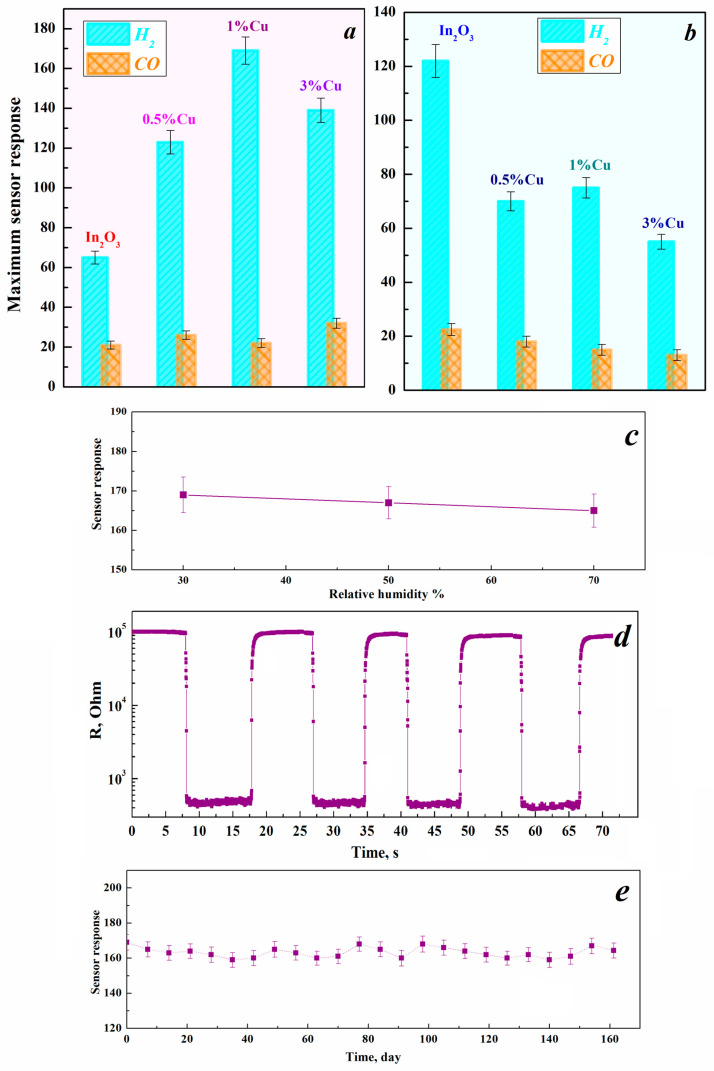
Concentration dependences of the maximum sensory response to 0.9% H_2_ and CO of Cu-In_2_O_3_ composites synthesized by the hydrothermal method using aqueous (**a**) and alcohol (**b**) solutions of indium and copper nitrates. Sensory response as a function of relative humidity (**c**), repeatability (**d**) and stability (**e**) of the 1% Cu-In_2_O_3_ composite synthesized by the hydrothermal method using aqueous solutions for 0.9% H_2_ detection.

Rhombohedral indium oxide synthesized from an alcoholic solution of indium nitrate exhibits a higher sensory response to hydrogen of 122 than cubic In_2_O_3_ of 65 (Figure 6). This trend may be attributed to several factors. Based on the structural data, *rh*-In_2_O_3_ has a higher specific surface area, and a larger number of oxygen vacancies, which are the centers of chemisorption of oxygen and the analyzed gas (Table 3). The introduction of copper into samples with a cubic structure increases the sensory response to both hydrogen and carbon monoxide (Figure 7a). In contrast, all copper additives into a rhombohedral phase of indium oxide produce a drop in sensory response to 0.9% H_2_ and CO irrespective of Cu concentration (Figure 7b). Such a trend was previously observed for hydrothermal ZnO-In_2_O_3_ systems in which maximum sensitivity was achieved for a composite containing cubic In_2_O_3_, and minimum for rhombohedral [12].

The maximum response to 0.9% H_2_ is demonstrated by the composite containing 1% Cu (Figure 7a). However, the composite containing 3% Cu had the highest sensor response for the detection of 0.9% CO. A similar effect is observed when introducing different concentrations of nickel ions into indium oxide [42]. The reason for the observed trends is not yet clear and requires further investigation. However, it is evident from the data presented in Figure 7a that the S_H2_/S_CO_ ratio, i.e., selectivity, is maximal for the 1% Cu-In_2_O_3_ composite. This is probably due to this composition having the largest specific surface area among the series of samples synthesized from aqueous solutions of indium and copper nitrates (Table 2). Note that selectivity is one of the characteristics of gas sensors that is the most difficult to achieve for conductometric-type sensors, especially for the detection of homogeneous gases such as hydrogen and carbon monoxide.

Increasing air humidity may negatively affect the gas sensing performance due to the adsorption of H_2_O instead of O_2_. However, only a slight decrease in response is observed from 30 to 70% RH for the 1% Cu-In_2_O_3_ composite in H_2_ detection (Figure 7c). Furthermore, this composite maintains signal strength over four cycles of 0.9% hydrogen fill/pump at the optimum operating temperature, demonstrating good repeatability (Figure 7d). Measurements over 5 months also showed that the value changed by less than 5–10%, indicating the long-term stability of the composite (Figure 7e).

The detection of reducing compounds, such as H_2_ and CO, by metal oxide sensors is based on the fact that their sensitive layer contains chemisorbed oxygen, which captures electrons from the conduction band of nanoparticles. In this case, various oxygen anion centers can form on the surface of these particles, the type of which depends on the chemisorption temperature [10]. In the generally accepted mechanism of operation of semiconductor oxide gas sensors, the determining factor is the change in the concentration of charge carriers caused by the chemical reactions of oxygen ions and the analyzed gas on the surface of nanoparticles. Increasing the number of defects, including oxygen vacancies, can provide more active sites for reactions on the surface of the sensitive layer. Alloying with Cu leads to a decrease in the oxygen content in both the cubic and rhombohedral lattices of In_2_O_3_, causing the formation of oxygen vacancies (see Table 3). This corresponds to the increase in O_V_ and the higher concentration of chemisorbed oxygen involved in the sensory reactions on the nanocomposite surface. This is associated with an increase in the sensory response to H2S or NO2 with the introduction of different concentrations of copper into the structure of cubic indium oxide [25,26]. However, an increase in the concentration of oxygen vacancies in metal oxides does not always lead to an increase in reactive forms of oxygen [42]. An increase in the concentration of chemisorbed oxygen O_C_ has a direct effect on gas-sensing characteristics. Based on the data obtained for composites with cubic structure, the concentrations of O_V_ and O_C_ increased significantly after the introduction of copper (Table 3). Although the addition of Cu to the corundum structure slightly increases the number of oxygen vacancies, the number of chemisorbed oxygen on the surface decreases (Table 3). The net result is a higher response to H_2_ and CO with copper introduction into the cubic lattice than the rhombohedral lattice.

In addition, the introduction of copper into indium oxide decreases the response time to the detected gases regardless of the phase state of indium oxide.

## 4. Conclusions

The structure, conductivity and sensory properties of indium oxide-based composites containing 1–3% copper oxide were studied. The composites were synthesized by hydrothermal method using solutions of indium and copper nitrates in water or ethanol. The composites synthesized from aqueous solutions exhibit a cubic structure of bixbyite In_2_O_3_ and are agglomerates of rectangular nanoparticles of non-uniform size. The nanoparticles of the composites obtained in alcohol have a spherical shape and contain rhombohedral In_2_O_3_ of the corundum type. All composites have a porous structure with a pore size in the range of 3.5–3.9 nm in samples synthesized in water, and 3–20 nm for those obtained in alcohol.

The composites based on rhombohedral In_2_O_3_ have a lower resistance than those based on the cubic structure of In_2_O_3_. The introduction of copper into different crystalline phases of In_2_O_3_ increases the film resistance. The sensory response to H_2_ and CO of cubic In_2_O_3_-based composites increases with the introduction of copper. A decrease in the optimal operating temperature is observed for both types of structures during the detection process.

## Figures and Tables

**Table 1:** XRD data for Cu-In_2_O_3_ composites.

Sample	Crystalline Phase	2θ, °	Lattice Parameters, nm	Particle Size, nm
**Hydrothermal samples (H_2_O)**				
In_2_O_3_	100% *c*-In_2_O_3_	30.6024 (222)	a = b = c = 1.011161	34.5
0.5% Cu-In_2_O_3_	100% *c*-In_2_O_3_	30.6078 (222)	a = b = c = 1.010771	33.9
1% Cu-In_2_O_3_	100% *c*-In_2_O_3_	30.6097 (222)	a = b = c = 1.010664	30.7
3% Cu-In_2_O_3_	100% *c*-In_2_O_3_	30.6118 (222)	a = b = c = 1.010602	30.3
**Hydrothermal samples (C_2_H_5_OH)**				
In_2_O_3_	100% *rh*-In_2_O_3_	31.0177(104)	a = b = 0.548671 c = 1.450863	24.6
0.5% Cu-In_2_O_3_	100% *rh*-In_2_O_3_	31.0194(104)	a = b = 0.548609c = 1.450558	24.5
1% Cu-In_2_O_3_	100% *rh*-In_2_O_3_	31.0198(104)	a = b = 0.548420c = 1.450442	23.6
3% Cu-In_2_O_3_	100% *rh*-In_2_O_3_	31.0208(104)	a = b = 0.548539c = 1.450134	21.4

**Table 2:** Parameter data for the mesoporous structure of synthesized composites.

Sample	S_BET_, m^2^/g	V_mes_, sm^3^/g	S_mes_, m^2^/g	d_mes_, nm	V_t_, sm^3^/g
**Hydrothermal samples (H_2_O)**					
In_2_O_3_	15.1	0.040	12.1	3.9	0.043
0.5% Cu-In_2_O_3_	16.6	0.040	13.8	3.9	0.042
1% Cu-In_2_O_3_	20	0.047	14.9	3.5	0.053
3% Cu-In_2_O_3_	19.8	0.040	15.7	3.5	0.045
**Hydrothermal samples (C_2_H_5_OH)**					
In_2_O_3_	23.1	0.070	16.3	3.2/19.3	0.074
0.5% Cu-In_2_O_3_	21.3	0.061	14.8	3.2/19.6	0.066
1% Cu-In_2_O_3_	23.2	0.062	15.6	3.2/18.9	0.068
3% Cu-97%In_2_O_3_	23.9	0.065	16.8	3.2/19	0.07

**Table 3:** Relationship between different forms of oxygen in Cu-In_2_O_3_ composites.

Sample	O_L_, %	O_V_, %	O_C_, %
**Hydrothermal samples (H_2_O)**			
In_2_O_3_	61.9	28.8	9.3
1% Cu-In_2_O_3_	55.1	34.3	10.6
**Hydrothermal samples (C_2_H_5_OH)**			
In_2_O_3_	62	31.8	6.2
1% Cu-In_2_O_3_	61.9	32.4	5.7

## Data Availability

Data is contained within the article.

## References

[1] Shaikh T., Jain S. (2023). ZnO nanostructure-based gas sensors: Critical review based on their synthesis and morphology towards various oxidizing and reducing gases. Curr. Nanomater..

[2] Gautam D., Gautam Y.K., Sharma K., Kumar A., Kumar A., Srivastava V., Singh B.P. (2024). Recent developments in SnO_2_ nanostructures inspired hydrogen gas sensors. Int. J. Hydrogen Energy.

[3] Li X., Fu L., Karimi-Maleh H., Chen F., Zhao S. (2024). Innovations in WO_3_ gas sensors: Nanostructure engineering, functionalization, and future perspectives. Heliyon.

[4] Shi Y., Li X., Sun X.F., Shao X., Wang H.Y. (2023). Strategies for improving the sensing performance of In_2_O_3_-based gas sensors for ethanol detection. J. Alloys Compd..

[5] Ikim M.I., Gerasimov G.N., Gromov V.F., Ilegbusi O.J., Trakhtenberg L.I. (2023). Synthesis, structural and sensor properties of nanosized mixed oxides based on In_2_O_3_ particles. Int. J. Mol. Sci..

[6] Morulane K.L., Swart H.C., Motaung D.E. (2024). A review on topical advancement and challenges of indium oxide based gas sensors: Future outlooks. J. Environ. Chem. Eng..

[7] Chen F., Yang M., Wang X., Song Y., Guo L., Xie N., Lu G. (2019). Template-free synthesis of cubic-rhombohedral-In_2_O_3_ flower for ppb level acetone detection. Sens. Actuators B Chem..

[8] Liang T.T., Kim D.S., Yoon J.W., Yu Y.T. (2021). Rapid synthesis of rhombohedral In_2_O_3_ nanoparticles via a microwave-assisted hydrothermal pathway and their application for conductometric ethanol sensing. Sens. Actuators B Chem..

[9] Cao E., Wu L., Zhang Y., Sun L., Yu Z., Nie Z. (2023). Hydrothermal synthesis of cubic-rhombohedral-In_2_O_3_ microspheres with superior acetone sensing performance. Appl. Surf. Sci..

[10] Trakhtenberg L.I., Ikim M.I., Ilegbusi O.J., Gromov V.F., Gerasimov G.N. (2023). Effect of nanoparticle interaction on structural, conducting and sensing properties of mixed metal oxides. Chemosensors.

[11] Sharma N., Choudhury S.P. (2024). Gas sensing using metal oxide semiconductor doped with rare earth elements: A review. Materials Sci. Eng. B.

[12] Ikim M.I., Gromov V.F., Gerasimov G.N., Spiridonova E.Y., Erofeeva A.R., Kurmangaleev K.S., Trakhtenberg L.I. (2023). Structure, conductivity, and sensor properties of nanosized ZnO-In_2_O_3_ composites: Influence of synthesis method. Micromachines.

[13] Shah S., Hussain S., Din S.T.U., Shahid A., Amu-Darko J.N.O., Wang M., Qiao G. (2024). A review on In_2_O_3_ nanostructures for gas sensing applications. J. Environ. Chem. Eng..

[14] Long H., Li Y., Chai K., Zeng W. (2024). Metal oxide semiconductor-based core-shell nanostructures for chemiresistive gas sensing: A review. Sens. Actuators B Chem..

[15] Shi C., Hou X., Guo R., Zhang W., Zhou Y. (2024). Starfish-like Zn doped In_2_O_3_ dendritic structure for superior triethylamine sensing by the facile co-precipitation method. Mater. Res. Bull..

[16] Li X., Xu X., Wang X., Li Y., Zhang B., Sun G., Wang Y. (2025). The multiple sensitization effects in La-doped In_2_O_3_ porous nanotubes enabling highly sensitive and selective detection of formaldehyde at low temperature. J. Alloys Compd..

[17] Wei D., Jiang W., Gao H., Chuai X., Liu F., Liu F., Lu G. (2018). Facile synthesis of La-doped In_2_O_3_ hollow microspheres and enhanced hydrogen sulfide sensing characteristics. Sens. Actuators B Chem..

[18] Yang Z., Chen X., Chen Q., Qu J., Guo Y., Zhou K., Ma X. (2025). Co ions doping enhances n-butanol sensing performance of In_2_O_3_ nanospheres. Sens. Actuators B Chem..

[19] Wang X., Li Y., Jin X., Sun G., Cao J., Wang Y. (2024). The effects of Co doping on the gas sensing performance of In_2_O_3_ porous nanospheres. Sens. Actuators B Chem..

[20] Yong P., Wang S., Zhang X., Pan H., Shen S. (2022). MOFs-derived Co-doped In_2_O_3_ hollow hexagonal cylinder for selective detection of ethanol. Chem. Phys. Lett..

[21] Wu W.J., Xu J.C., Hong B., Li J., Zeng Y.X., Peng X.L., Wang X.Q. (2025). Highly-enhanced gas-sensing performance of metal-doped In_2_O_3_ microtubes from acceptor doping and double surface adsorption. Mater. Sci. Eng. B.

[22] Yan P., Hou W., Wang M., Li Y., Ge C., Zhang Z., Bai L. (2025). Light-activated 3DOM Zn-doped In_2_O_3_ for room-temperature ppb-level NO_2_ detection. Sens. Actuators B Chem..

[23] Kulkarni S.C., Salunke V.T., Naeem S., Patil A.V. (2025). Enhanced structural, optical, and gas sensing properties of Zn-doped In_2_O_3_ nanomaterial synthesized via sol-gel technique. Ceram. Int..

[24] Ye F., Cai X.M., Zhong X., Tian X.Q., Jing S.Y., Huang L.B., Liang G.X. (2014). The electrical and optical properties of Cu-doped In_2_O_3_ thin films. Thin Solid Films.

[25] Hu X., Tian L., Sun H., Wang B., Gao Y., Sun P., Lu G. (2015). Highly enhanced NO_2_ sensing performances of Cu-doped In_2_O_3_ hierarchical flowers. Sens. Actuators B Chem..

[26] Zhang Y., Han S., Wang M., Liu S., Liu G., Meng X., Qiao G. (2022). Electrospun Cu-doped In_2_O_3_ hollow nanofibers with enhanced H_2_S gas sensing performance. J. Adv. Ceram..

[27] Lemos S.C., Romeiro F.C., de Paula L.F., Gonçalves R.F., de Moura A.P., Ferrer M.M., Lima R.C. (2017). Effect of Er^3+^ ions on the phase formation and properties of In_2_O_3_ nanostructures crystallized upon microwave heating. J. Solid State Chem..

[28] Lemos S.C.S., Nossol E., Ferrari J.L., Gomes E.O., Andres J., Gracia L., Lima R.C. (2019). Joint theoretical and experimental study on the La doping process in In_2_O_3_: Phase transition and electrocatalytic activity. Inorg. Chem..

[29] Li P., Cai C., Cheng T., Huang Y. (2017). Hydrothermal synthesis and Cl_2_ sensing performance of porous-sheets-like In_2_O_3_ structures with phase transformation. RSC Adv..

[30] Ikim M.I., Gerasimov G.N., Erofeeva A.R., Gromov V.F., Ilegbusi O.J., Trakhtenberg L.I. (2024). Cobalt doped cubic and rhombohedral In_2_O_3_: The role of crystalline phase of indium oxide in sensor response to hydrogen. Chem. Phys. Lett..

[31] Roso S., Vilic T., Urakawa A., Llobet E. (2016). Gas sensing properties of In_2_O_3_ cubes prepared by a hydrothermal method. Procedia Eng..

[32] Van Tong P., Minh L.H., Van Duy N., Hung C.M. (2021). Porous In_2_O_3_ nanorods fabricated by hydrothermal method for an effective CO gas sensor. Mater. Res. Bull..

[33] Gerasimov G.N., Ikim M.I., Gromov V.F., Ilegbusi O.J., Trakhtenberg L.I. (2021). Chemical modification of impregnated SnO_2_-In_2_O_3_ nanocomposites due to interaction of sensor components. J. Alloys Compd..

[34] Muruganandham M., Lee G.J., Wu J.J., Levchuk I., Sillanpaa M. (2013). By-product assisted hydrothermal synthesis of InOOH microflower composed of nanosheets. Mater. Lett..

[35] Wu L., Cao E., Zhang Y., Sun L., Sun B., Yu Z. (2023). La and Fe co-doped walnut-like cubic-rhombohedral-In_2_O_3_ for highly sensitive and selective detection of acetone vapor. Mater. Lett..

[36] Li L., Hong D., Liu B., Su T., Yang X., Yue L., Zhang W. (2025). Porous Zn-doped In_2_O_3_ nanobelts for ppb level acetone sensing at low operating temperature. Sens. Actuators B Chem..

[37] Yan T., Wang X., Long J., Lin H., Yuan R., Dai W., Fu X. (2008). Controlled preparation of In2O3, InOOH and In(OH)_3_ via a one-pot aqueous solvothermal route. New J. Chem..

[38] Yu D., Yu S.H., Zhang S., Zuo J., Wang D., Qian Y.T. (2003). Metastable hexagonal In_2_O_3_ nanofibers templated from InOOH nanofibers under ambient pressure. Adv. Funct. Mater..

[39] Yang H., Wang S., Yang Y. (2012). Zn-doped In_2_O_3_ nanostructures: Preparation, structure and gas-sensing properties. Cryst. Eng. Comm..

[40] Liu J., Zhang L., Fan J., Zhu B., Yu J. (2021). Triethylamine gas sensor based on Pt-functionalized hierarchical ZnO microspheres. Sens. Actuators B Chem..

[41] Kou X., Meng F., Chen K., Wang T., Sun P., Liu F., Lu G. (2020). High-performance acetone gas sensor based on Ru-doped SnO_2_ nanofibers. Sens. Actuators B Chem..

[42] Qin W., Lu B., Xu X., Shen Y., Meng F. (2024). Metal organic framework-derived porous Ni-doped In_2_O_3_ for highly sensitive and selective detection to hydrogen at low temperature. Sens. Actuators B Chem..

